# Rare papillary renal neoplasm with reverse polarity: A case report and review of the literature

**DOI:** 10.3389/fonc.2023.1101268

**Published:** 2023-03-17

**Authors:** Xi Tu, Xiyao Zhuang, Qiong Chen, Wei Wang, Chaoyou Huang

**Affiliations:** ^1^ Department of Urology, Chengdu Second People’s Hospital, Chengdu, Sichuan, China; ^2^ Department of Internal Medicine, Chengdu Shuangliu Hospital of Traditional Chinese Medicine, Chengdu, Sichuan, China; ^3^ Department of Pathology, Chengdu Second People’s Hospital, Chengdu, Sichuan, China

**Keywords:** papillary renal neoplasm with reverse polarity (PRNRP), papillary renal cell carcinomas, GATA3, *KRAS*, histopathology

## Abstract

Papillary renal neoplasm with reverse polarity (PRNRP) is a rare renal tumour and was newly named in 2019. This study reported a case of a 30-year-old female patient with a left renal tumour without any clinical symptoms and whose CT scan of her left kidney showed a mass of 2.6 cm×2.3 cm, which was considered to be renal clear cell carcinoma. Laparoscopic partial nephrectomy was performed, and histopathology and immunohistochemistry confirmed papillary renal neoplasm with reverse polarity, which had unique clinicopathological features, immunophenotype, *KRAS* gene mutation and relatively indolent biological behaviour. As newly diagnosed cases, rigorous and regular follow-up is necessary. In addition, a literature review was performed from 1978 to 2022, and 97 cases of papillary renal neoplasms with reverse polarity were identified and analysed.

## Introduction

Papillary renal neoplasm with reverse polarity is a newly reported papillary renal tumour that accounts for approximately 4% of all previously diagnosed papillary renal cell carcinomas (PRCC) (1). This tumour was initially classified as papillary renal cell carcinoma, but it has unique morphological features and a better prognosis. In 2019, AI-Obaidy et al. first diagnosed papillary renal neoplasm with reverse polarity and proved that it was different from papillary renal cell carcinoma in pathological morphology, immunohistochemistry and chromosomal features (2). Because PRNRP is rare, it is easily misdiagnosed preoperatively as other types of renal tumours. Surgeons often choose the appropriate surgical method based on their experience in the treatment of common renal tumours. The key to the treatment is to completely remove the tumour. Current data suggest a good prognosis after resection of PRNRP (1, 2); however, the long-term outcome is unclear, and regular follow-up is necessary. Here, we reported a case of papillary renal neoplasm with reverse polarity and reviewed the relevant literature to further understand the clinical features, pathology, treatment and prognosis of PRNRP, and to strengthen the awareness of this rare disease.

## Case presentation

**Figure 1:**
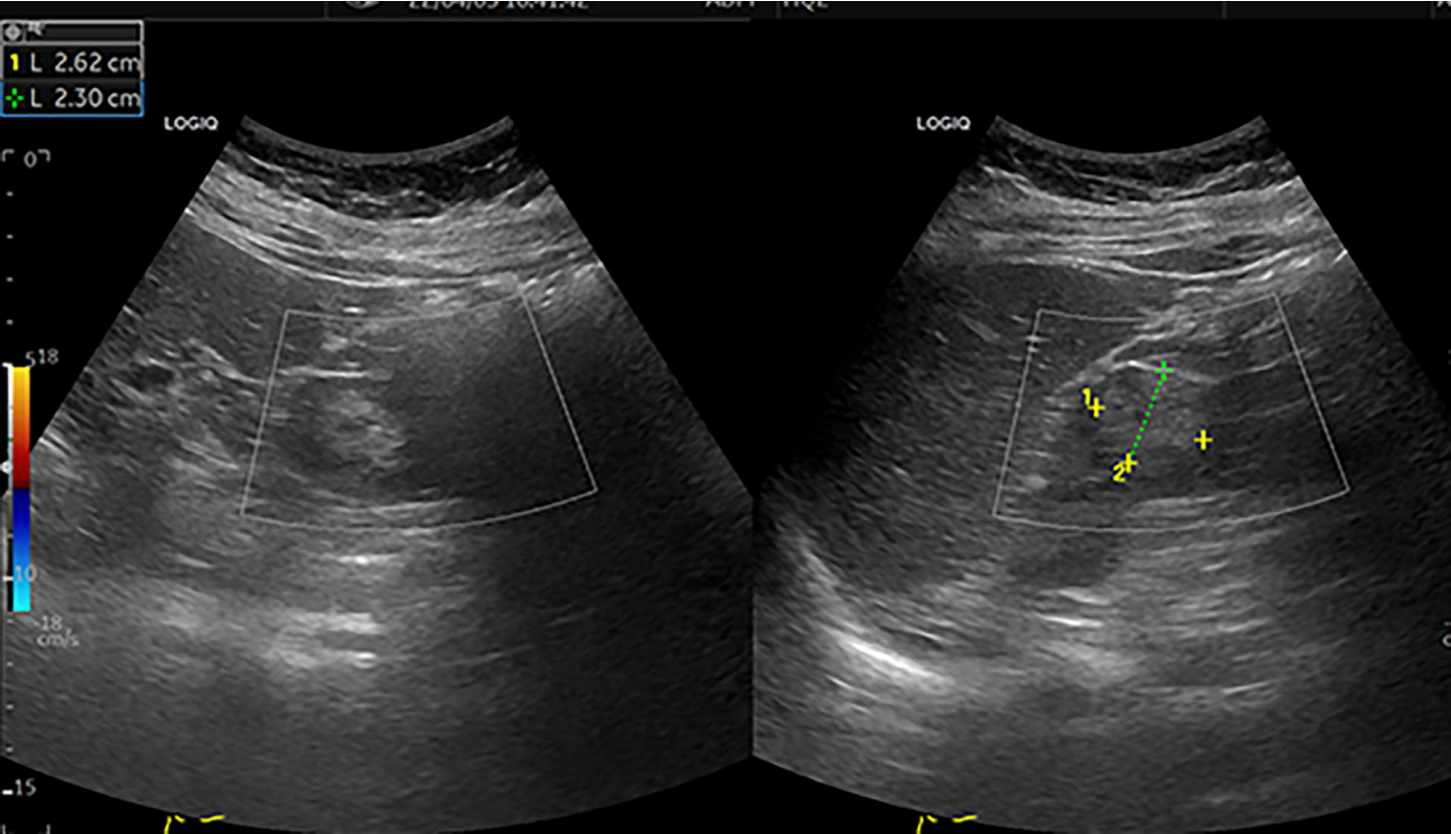
Colour ultrasound. This image shows a moderate echogenic mass of approximately 2.6 cm×2.3 cm in the middle and upper parts of the left kidney, with a clear boundary and regular shape [yellow^+^ and green^+^]. CDFI: no blood flow signal in the mass.

**Figure 2:**
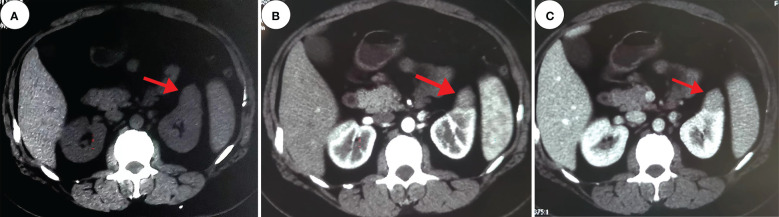
Computed tomography. These images show a 2.4 cm×2.2 cm equal-density mass in the anterior upper part of the left kidney, with poorly defined margins and a calcium density shadow in the mass [**(A)**, red arrow]. The enhancement scan shows inhomogeneous enhancement [**(B, C)**, red arrow].

**Figure 3:**
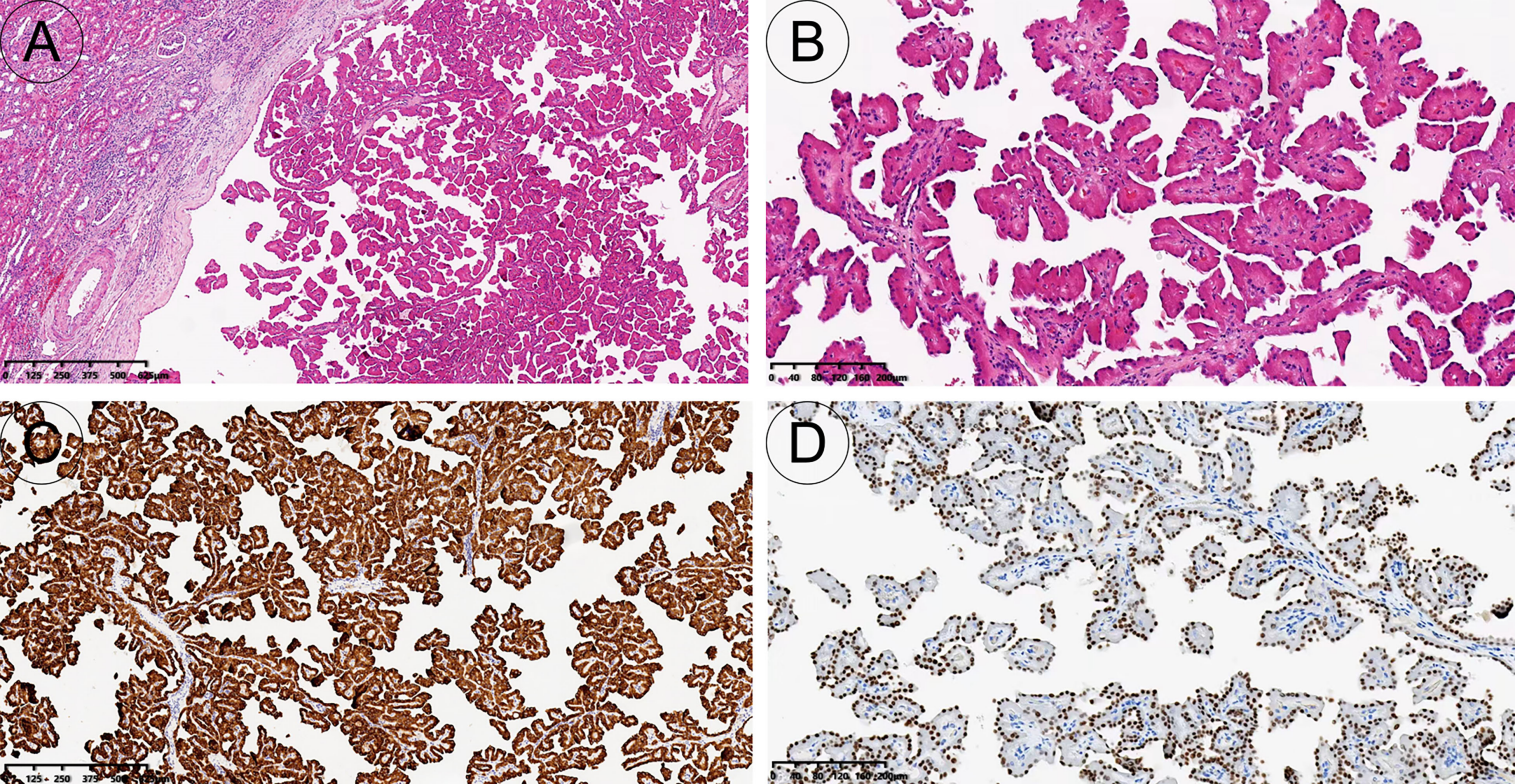
Pathology and immunohistochemistry. The tumour is well demarcated and has a complex branched papillary structure with a fibrous vascular axis [H&E, 40x **(A)**]; The surface is covered with a monolayer of cuboidal or columnar cells, with eosinophilic cytoplasm and characteristic nuclei located at the top of the cytoplasm away from the basement membrane [H&E, 100x **(B)**]. The tumour cells expressed keratin 7 diffusely and strongly [immunohistochemistry, 40x **(C)**]; GATA3 was diffusely expressed in tumour nuclei [immunohistochemistry, 100x **(D)**].

A 30-year-old female patient was admitted to the hospital with a left renal mass found on physical examination. During the course of the disease, there was no low back pain, haematuria, frequent urination and pain, dizziness, palpitations, fever or chills. The patient had not received any specific treatment previously and followed a healthy diet and lifestyle and had no family history of the disease or similar diseases. The patient’s vital signs were normal. There was no swelling, tenderness or pain induced by tapping over either kidney area. Urological ultrasound showed a moderate echogenic mass of approximately 2.6 cm×2.3 cm in the middle and upper parts of the left kidney, with a clear boundary and regular shape and no blood flow signal (**Figure 1**). Chest CT showed no abnormalities. Abdominal CT showed a left renal mass, and renal clear cell carcinoma was considered (**Figure 2**). The preoperative diagnosis was a left renal mass, and the patient underwent laparoscopic partial left nephrectomy. The tumour capsule was intact in the resected specimen, and brown fish-like tissue was observed after a longitudinal incision of the tumour. Histopathological studies of the resected tumour revealed the tumour was well demarcated and had a complex branched papillary structure with a fibrous vascular axis, and the papillary surface was covered with a monolayer of cuboidal or columnar cells, with eosinophilic cytoplasm and characteristic nuclei located at the top of the cytoplasm away from the basement membrane (**Figures 3A, B**). Immunohistochemical studies of the tumour showed that the lesion was positive for the expression of GATA3, KRT7, p504s, EMA, PAX-2, PAX-8, SHDB, Ki-67 and TTF-1 and negative for vimentin, CD5, CD10, WT-1, CAIX, TFE-3, HMB-45, CD117, ALK and TG (**Figures 3C, D**). Histomorphology and immunophenotype were consistent with papillary renal neoplasm with reverse polarity. The patient declined further molecular genetic testing for financial reasons. The patient did not receive any treatment for 7 months after the operation, and there was no recurrence or metastasis.

## Systematic review of literature

The PubMed database was searched for case reports and case series of papillary renal cell carcinoma and papillary renal neoplasm with reverse polarity published between 1978 and 2022. Using the following keywords: (oncocytic papillary renal cell carcinoma) or (oncocytic PRCC) or (papillary renal neoplasm with reverse polarity) or (PRNRP), 403 results were retrieved. After removing unrelated studies, 11 publications describing 97 cases were finally identified (**Table 1**). The review series included 97 patients (56 men and 41 women) with a definite diagnosis of papillary renal tumour with reverse polarity. The evaluation showed that 31 cases of PRNRP occurred in the left kidney, and 43 cases occurred in the right kidney. The age of PRNRP patients ranged from 35 to 82 years, with an average age of 62.2 years. The diameter of PRNRP ranged from 0.8 to 8.5 cm, with an average diameter of 2.1 cm. Most tumours have no clinical symptoms and are diagnosed incidentally during imaging examination. The World Health Organization (WHO)/International Society of Urological Pathology (ISUP) showed low nuclear grade (13), and most of the reported PRNRP cases were staged as pT1. Among them, 52 patients underwent laparoscopic partial nephrectomy, 10 patients underwent laparoscopic radical nephrectomy, and 2 patients underwent renal biopsy, all of which were confirmed as PRNRP by histopathology. Seventy-four of 97 patients were followed up from 1 month to 222 months, and no tumour recurrence during the follow-up period.

**Table 1:** Clinicopathologic Features of 97 Patients With PRNRP in the Literature.

Case NO	First author, year	Sex	Age(y)	Symptoms	Location	Size(cm)	Surgery	Stage	Follow-up (mo)	Recurrence/ Metastasis
1	Al-Obaidy et al., 2019 (2)	M	50	NA	Left	2.5	PN	pT1a	NA	NA
2	Al-Obaidy et al., 2019 (2)	F	70	NA	Left	1.2	RN	pT1a	NA	NA
3	Al-Obaidy et al., 2019 (2)	F	72	NA	Left	2.3	Renal biopsy	pT1a	7	No
4	Al-Obaidy et al., 2019 (2)	F	75	NA	Right	1.0	PN	pT1a	44	No
5	Al-Obaidy et al., 2019 (2)	M	69	NA	Right	1.0	PN	pT1a	16	NA
6	Al-Obaidy et al., 2019 (2)	M	80	NA	Left	1.5	PN	pT1a	NA	NA
7	Al-Obaidy et al., 2019 (2)	F	76	NA	Left	1.5	Renal biopsy	pT1a	2	No
8	Al-Obaidy et al., 2019 (2)	F	77	NA	Right	1.0	RN	pT1a	30	No
9	Al-Obaidy et al., 2019 (2)	F	54	NA	Right	1.0	RN	pT1a	48	No
10	Al-Obaidy et al., 2019 (2)	M	66	NA	Right	1.0	RN	pT1a	80	No
11	Al-Obaidy et al., 2019 (2)	F	58	NA	Right	1.1	RN	pT1a	1	No
12	Al-Obaidy et al., 2019 (2)	F	46	NA	Left	3.0	PN	pT1a	1	No
13	Al-Obaidy et al., 2019 (2)	M	54	NA	Right	1.8	PN	pT1a	67	No
14	Al-Obaidy et al., 2019 (2)	M	66	NA	Right	3.0	PN	pT1a	17	No
15	Al-Obaidy et al., 2019 (2)	M	50	NA	Right	1.1	PN	pT1a	22	No
16	Al-Obaidy et al., 2019 (2)	F	64	NA	Right	3.0	RN	pT1a	NA	No
17	Al-Obaidy et al., 2019 (2)	M	75	NA	Right	0.8	RN	pT1a	7	No
18	Al-Obaidy et al., 2019 (2)	M	50	NA	Right	2.3	PN	pT1a	222	No
19	Tong et al., 2020 (3)	M	56	Incidental detection	Right	2.7	PN	pT1a	5	No
20	Tong et al., 2020 (3)	M	51	Incidental detection	Left	2.8	PN	pT1a	5	No
21	Tong et al., 2020 (3)	M	61	Incidental detection	Left	2.5	RN	pT1a	17	No
22	Tong et al., 2020 (3)	M	42	Incidental detection	Right	2.0	PN	pT1a	22	No
23	Tong et al., 2020 (3)	M	60	Incidental detection	Left	4.5	PN	pT1b	36	No
24	Tong et al., 2020 (3)	M	78	Incidental detection	Right	1.0	RN	pT1a	38	No
25	Tong et al., 2020 (3)	F	52	Haematuria	Left	1.5	PN	pT1a	NA	NA
26	Tong et al., 2020 (3)	F	59	Incidental detection	Right	3.0	PN	pT1a	NA	NA
27	Tong et al., 2020 (3)	M	42	Lower back pain	Left	1.5	PN	pT1a	35	No
28	Tong et al., 2020 (3)	M	63	Incidental detection	Right	1.9	PN	pT1a	57	No
29	Tong et al., 2020 (3)	F	50	NA	Right	3.2	PN	pT1a	24	No
30	Tong et al., 2020 (3)	M	60	NA	Left	1.5	PN	pT1a	23	No
31	Tong et al., 2020 (3)	F	79	NA	Right	2.5	PN	pT1a	41	No
32	Zhou et al., 2020 (4)	F	69	NA	Left	2.5	NA	pT1a	48	No
33	Zhou et al., 2020 (4)	M	48	NA	Right	2.2	NA	pT1a	24	No
34	Zhou et al., 2020 (4)	M	65	NA	Left	8.0	NA	pT1a	7	No
35	Zhou et al., 2020 (4)	M	61	NA	Right	2.5	NA	pT1a	3	No
36	Zhou et al., 2020 (4)	F	74	NA	Right	1.2	NA	pT1a	50	No
37	Zhou et al., 2020 (4)	F	58	NA	Right	2.5	NA	pT1a	65	No
38	Zhou et al., 2020 (4)	M	54	NA	Right	1.5	NA	pT1a	3	No
39	Kim et al., 2020 (5)	F	64	NA	Left	1.3	PN	pT1a	200	No
40	Kim et al., 2020 (5)	M	59	NA	Right	1.0	PN	pT1a	15	No
41	Kim et al., 2020 (5)	F	61	NA	Right	2.0	PN	pT1a	7	No
42	Kim et al., 2020 (5)	F	70	NA	Right	1.3	PN	pT1a	133	No
43	Kim et al., 2020 (5)	M	72	NA	Right	0.9	PN	pT1a	53	No
44	Kim et al., 2020 (5)	M	52	NA	Left	1.2	PN	pT1a	79	No
45	Kim et al., 2020 (5)	M	36	NA	Left	1.5	PN	pT1a	86	No
46	Kim et al., 2020 (5)	F	69	NA	Left	2.5	PN	pT1a	60	No
47	Kim et al., 2020 (5)	M	52	NA	Left	1.5	PN	pT1a	84	No
48	Kim et al., 2020 (5)	M	60	NA	Right	1.5	PN	pT1a	72	No
49	Kim et al., 2020 (5)	M	54	NA	Left	3.0	RN	pT1a	47	No
50	Kim et al., 2020 (5)	F	58	NA	Left	1.3	PN	pT1a	34	No
51	Kim et al., 2020 (5)	M	55	NA	Left	1.8	PN	pT1a	46	No
52	Kim et al., 2020 (5)	M	61	NA	Right	1.0	PN	pT1a	99	No
53	Kim et al., 2020 (5)	M	66	NA	Left	1.5	PN	pT1a	59	No
54	Kim et al., 2020 (5)	M	64	NA	Right	1.2	PN	pT1a	21	No
55	Kim et al., 2020 (5)	F	57	NA	Left	0.9	PN	pT1a	16	No
56	Kim et al., 2020 (5)	F	56	NA	Left	1.5	PN	pT1a	9	No
57	Kim et al., 2020 (5)	F	51	NA	Left	2.7	PN	pT1a	164	No
58	Kim et al., 2020 (5)	M	59	NA	Right	1.7	PN	pT1a	55	No
59	Kim et al., 2020 (5)	M	63	NA	Right	1.7	PN	pT1a	91	No
60	Kim et al., 2020 (5)	M	61	NA	Left	1.9	PN	pT1a	32	No
61	Kim et al., 2020 (5)	M	68	NA	Right	1.5	PN	pT1a	30	No
62	Kim et al., 2020 (5)	F	74	NA	Right	2.2	PN	pT1a	40	No
63	Kim et al., 2020 (5)	M	61	NA	Left	1.6	PN	pT1a	13	No
64	Kim et al., 2020 (5)	F	51	NA	Right	2.0	PN	pT1a	21	No
65	Kim et al., 2020 (5)	F	77	NA	Right	3.0	PN	pT1a	17	No
66	Kim et al., 2020 (5)	M	61	NA	Right	1.7	PN	pT1a	16	No
67	Kim et al., 2020 (5)	M	67	NA	Left	2.3	PN	pT1a	11	No
68	Kim et al., 2020 (5)	M	60	NA	Right	5.8	PN	pT1a	29	No
69	Lee et al., 2020 (6)	M	67	NA	Right	4.5	NA	pT1b	6	No
70	Song et al., 2020 (7)	M	54	Incidental detection	Right	2.3	PN	pT1a	6	No
71	Kiyozawa et al., 2021 (8)	F	48	Incidental detection	NA	3.0	NA	pT1a	NA	No
72	Kiyozawa et al., 2021 (8)	F	58	Incidental detection	NA	1.3	NA	pT1a	NA	No
73	Kiyozawa et al., 2021 (8)	M	66	Incidental detection	NA	1.4	NA	pT1a	NA	No
74	Kiyozawa et al., 2021 (8)	F	65	Incidental detection	NA	1.2	NA	pT1a	NA	No
75	Kiyozawa et al., 2021 (8)	M	47	Incidental detection	NA	1.2	NA	pT1a	NA	No
76	Kiyozawa et al., 2021 (8)	F	82	Incidental detection	NA	1.0	NA	pT1a	NA	No
77	Kiyozawa et al., 2021 (8)	F	77	Incidental detection	NA	3.0	NA	pT1a	NA	No
78	Kiyozawa et al., 2021 (8)	M	78	Incidental detection	NA	5.0	NA	pT1b	NA	No
79	Kiyozawa et al., 2021 (8)	M	72	Incidental detection	NA	1.4	NA	pT1a	NA	No
80	Kiyozawa et al., 2021 (8)	F	68	Incidental detection	NA	2.0	NA	pT1a	NA	No
81	Kiyozawa et al., 2021 (8)	M	66	Incidental detection	NA	1.7	NA	pT1a	NA	No
82	Kiyozawa et al., 2021 (8)	F	79	Incidental detection	NA	1.0	NA	pT1a	NA	No
83	Kiyozawa et al., 2021 (8)	M	73	Incidental detection	NA	1.7	NA	pT1a	NA	No
84	Kiyozawa et al., 2021 (8)	F	75	Incidental detection	NA	3.0	NA	pT1a	NA	No
85	Kiyozawa et al., 2021 (8)	M	66	Incidental detection	NA	1.4	NA	pT1a	NA	No
86	Pivovarcikova et al., 2021 (9)	F	56	NA	NA	2.0	NA	pT1a	NA	NA
87	Pivovarcikova et al., 2021 (9)	F	71	NA	Left	2.1	NA	pT1a	17	No
88	Pivovarcikova et al., 2021 (9)	M	67	NA	Right	4.5	NA	pT1b	6	No
89	Wei et al., 2022 (10)	F	67	NA	NA	1.0	NA	pT1a	23	No
90	Wei et al., 2022 (10)	F	40	NA	NA	8.5	NA	pT2a	141	No
91	Wei et al., 2022 (10)	M	49	NA	NA	2.0	NA	pT1a	110	No
92	Wei et al., 2022 (10)	M	71	NA	NA	3.5	NA	pT1a	33	No
93	Wei et al., 2022 (10)	F	74	NA	NA	3.5	NA	pT1a	20	No
94	Wei et al., 2022 (10)	M	61	NA	NA	0.9	NA	pT1a	12	No
95	Wei et al., 2022 (10)	M	69	NA	NA	2.3	NA	pT1a	12	No
96	Zhang et al., 2022 (11)	M	71	Incidental detection	Left	1.8	PN	NA	NA	NA
97	Wang et al., 2022 (12)	M	35	Incidental detection	Right	1.7	PN	NA	6	No

F, female; M, male; y, years old; N/A, not available; RN, radical nephrectomy; PN, partial nephrectomy.

## Discussion

Papillary renal neoplasm with reverse polarity is a rare type of renal neoplasm reported recently. In 2003, Allory et al. found that some papillary renal cell carcinomas had a good prognosis and called it “oncocytoid-type papillary renal cell carcinoma” (14). Lefevre et al. named it “oncocytic papillary renal cell carcinoma”, and this term was widely used in 2005 (15). In 2017, Saleeb et al. classified papillary renal cell carcinoma into 4 types based on immunohistochemical and molecular phenotypes and proposed the term “low-grade eosinophilic papillary renal cell carcinoma, type 4” (16). In 2019, AI-Obaidy et al. named this tumour papillary renal neoplasm with reverse polarity for the first time and proved that it was different from papillary renal cell carcinoma type I and type II in terms of pathological morphology, immunophenotype and chromosomal characteristics (2). Subsequently, 89 additional cases of PRNRP were reported, and 8 patients did not include detailed clinical data (1) and were therefore not included in **Table 1**.

According to the study reported by AI-Obaidy et al., the incidence of PRNRP was similar in males and females, and the age ranged from 46 to 80 years, with an average age of 64 years (2). However, our systematic review showed that the incidence of PRNRP was slightly higher in males than in females, with an average age of 62.2 years. In terms of age, the evaluation indicated that the youngest patient in the past was 35 years old, while the present patient was 30 years old, which is the youngest patient identified to date. Previous data indicated that the tumour size was 3.0 cm or less, with an average of 1.6 cm. The evaluation results showed that the average tumour diameter of 97 patients with PRNRP was 2.1 cm (range: 0.8-8.5 cm). PRNRPs are usually asymptomatic, and often are discovered incidentally on imaging. Although our evaluation shows that the majority of PRNRPs are small in size, as they gradually grow, they may compress surrounding organs, impair kidney function, and even rupture bleeding,etc.

PRNRP usually has no specific clinical symptoms, thus posing significant preoperative diagnostic challenges. Imaging examinations do not provide much diagnostic information because of their rarity. At present, there is a lack of literature reports on the imaging features of PRNRP, and more data need to be collected and further explored. Histopathology and immunohistochemistry are the gold standard for the diagnosis of PRNRP. Chang et al. proposed the following four diagnostic criteria: (I) Mainly protruding thin papillary or tubular papillary growth; (II) Focal or diffuse interstitial vitrification; (III) Eosinophilic fine granular cytoplasm; (IV) The tumour nuclei were neatly arranged on the top of the cytoplasm far away from the basement membrane, showing the characteristics of “reverse polarity”, with the same size and low nuclear grade (1). This patient was a 30-year-old female who was considered to be diagnosed with clear cell carcinoma before surgery and underwent laparoscopic partial nephrectomy. The postoperative pathological diagnosis was PRNRP, and the clinical stage was pT1. The patient did not receive any treatment and did not have any discomfort for 7 months after the operation.

Immunophenotypically, PRCC strongly expressed vimentin and p504s, but did not express GATA3 and 34βE12. PRNRP strongly expressed GATA3 and KRT7, and expressed p504s to varying degrees, and could express 34βE12, but could not express vimentin. This tumor strongly expressed KRT7 and partially expressed p504s, but did not express CD10, which was consistent with the PRCC phenotype. However, unlike other PRCC subtypes, PRNRP typically expresses GATA3. PRNRP does not express vimentin, CD10, CAIX, CD117, TFE-3, ALK, etc, which is helpful for differential diagnosis from other rare types of renal cell carcinoma. The low proliferation index of Ki-67 suggests that it has a good prognosis. The above immunophenotypes were consistent with those reported in the literature (2), supporting the diagnosis of PRNRP in this case. In addition, 7 cases of PRNRP reported by Zhou et al. were all positive for 34βE12 except for the specific expression of GATA3 (4), which was not recorded in other studies. The results provide new insights into the diagnosis and even treatment of papillary renal neoplasms with reverse polarity.

Recent studies have shown that PRNRP has high-frequency *KRAS* gene mutations; the *KRAS* mutation rate was found in 85% of the tested cases, and *KRAS* gene mutations in PRNRP were concentrated in the exon 2 codon 12 (3, 10). Among them, the G12 V missense mutation was the hotspot mutation (mutation rate was 33.3%-75.0%), followed by G12D (0-30.7%), G12R (3.8%-25.0%) and G12C (0-11.1%), the BRAF V600R mutation was detected in one *KRAS* wild-type case (3, 5, 17). Fluorescence *in situ* hybridization (FISH) analysis showed that 32% (14/44) of papillary renal tumours with reverse polarity had abnormalities in chromosomes 7 and 17, and only 2 cases (2/44) had chromosome Y deletion (10), which further proved that it was similar but not identical to classic PRCC. In this study, because the patient refused to undergo molecular genetic testing, we could not further understand whether the patient had gene mutations and chromosomal variations. Therefore, PRNRP has unique clinicopathological features, immunophenotypes, and *KRAS* gene mutations and can be clinically differentiated from common papillary renal cell carcinoma, renal papillary adenoma, clear cell papillary renal cell carcinoma, and Xp11.2 translocation-associated renal cell carcinoma.

To date, there is no consensus on the optimal treatment strategy for PRNRP. The preferred treatment for any nonmetastatic, solid renal mass is surgical resection, preferably using a minimally invasive approach (18). For localized renal tumours, surgical treatment mainly includes radical nephrectomy (RN) and partial nephrectomy (PN). Nephron sparing partial nephrectomy is recommended for certain patients, and a negative surgical margin should be achieved while removing the renal mass. Compared with radical nephrectomy, partial nephrectomy can preserve the normal renal parenchyma while removing the tumour, reduce the incidence of long-term renal insufficiency, reduce the incidence of cardiovascular events, and improve the quality of life of patients with renal tumours (19–22). Other options for treating renal masses less than 3 cm include thermal ablation, cryoablation, and radiofrequency ablation. Renal mass biopsy should be performed in all patients receiving these regimens to facilitate histological diagnosis and guide subsequent treatment and follow-up. However, patients should be advised that these treatment options increase the risk of local recurrence or tumour persistence (18). At present, there is no literature report on the treatment of PRNRP by various ablations, and further studies are needed to verify its effect. Active monitoring is an acceptable option for some patients with renal masses less than 2 cm (grade C). It is suitable for elderly patients with serious complications or short life expectancy. However, continuous imaging must be performed to monitor changes in renal tumour size. Patients and their families need to understand the risks of active surveillance. For patients who choose active surveillance, renal mass biopsy is recommended for further risk factor stratification (18). If the benefit of intervention exceeds the benefit of active surveillance, active treatment should be chosen. Combined with a literature review, PRNRP is usually treated by partial nephrectomy or radical nephrectomy, and the treatment effect is good. Urologists can choose the appropriate treatment according to the specific situation of patients and their own clinical experience. Although PRNRP has a good prognosis, the current data are insufficient to draw conclusions about the long-term efficacy of treatment for this tumour, and regular follow-up is necessary. In our study, the patient had no clinical symptoms, no abnormalities on chest CT, no surrounding organ infiltration or regional lymph node enlargement on abdominal CT, so the diagnosis of localized renal tumor was considered. After we actively communicated with the patient about the treatment plan, the patient underwent laparoscopic partial nephrectomy to remove the tumour and absence of recurrence at follow-up, and renal ultrasound or CT examination is necessary in the future.

## Patient perspective

Kidney tumor had brought me great trouble and anxiety, affecting my daily life. After talking to my doctor, I underwent a laparoscopic partial nephrectomy to remove the tumor. When histopathology and immunohistochemistry confirmed PRNRP, my fears and concerns disappeared. I achieved physical and psychological healing. I think I have been treated successfully. I will follow the doctor’s advice for regular follow-up in the future.

## Conclusion

PRNRP is a newly recognized low-grade renal tumour with relatively indolent biological behaviour. Its pathological morphology, immunophenotype and molecular genetic changes are different from those of classical PRCC type 1 and type 2. It may be a special subtype of PRCC, which has not yet been classified into the WHO (2016) classification of renal tumours (23). However, PRNRP is a provisional subtype of papillary RCC in WHO 2022 but has not yet been incorporated into an independent histological type or subtype (24). Urological surgeons should recognize this rare disease to distinguish it from other renal tumours. Due to the rarity of this tumour, its pathogenesis and histological origin still need to be further improved, and more cases and follow-up data need to be accumulated to further explore its biological behaviour. Therefore, it is of positive clinical significance to distinguish PRNRP from papillary renal cell carcinoma for targeted therapy.

## Data availability statement

The original contributions presented in the study are included in the article/supplementary material. Further inquiries can be directed to the corresponding author.

## Ethics statement

The studies involving human participants were reviewed and approved by the Ethics Committee of Chengdu Second People’s Hospital. The patients/participants provided their written informed consent to participate in this study. Written informed consent was obtained from the individual(s) for the publication of any potentially identifiable images or data included in this article. Written informed consent was obtained from the participant/patient(s) for the publication of this case report.

## Author contributions

XT was the patient’s urologists, reviewed the literature and contributed to manuscript drafting. XZ reviewed the literature and prepared figures. WW, QC and CH were responsible for the revision of the manuscript for important intellectual content. All authors issued final approval for the version to be submitted. All authors contributed to the article and approved the submitted version.

## References

[1] Chang HY Hang JF Wu CY Lin TP Chung HJ Chang YH. Clinicopathological and molecular characterisation of papillary renal neoplasm with reverse polarity and its renal papillary adenoma analogue. Histopathology (2021) 78(7):1019–31. doi: 10.1111/his.14320 33351968

[2] Al-Obaidy KI Eble JN Cheng L Williamson SR Sakr WA Gupta N. Papillary renal neoplasm with reverse polarity: A morphologic, immunohistochemical, and molecular study. Am J Surg Pathol (2019) 43(8):1099–111. doi: 10.1097/PAS.0000000000001288 31135486

[3] Tong K Zhu W Fu H Cao F Wang S Zhou W. Frequent KRAS mutations in oncocytic papillary renal neoplasm with inverted nuclei. Histopathology (2020) 76(7):1070–83. doi: 10.1111/his.14084 31997427

[4] Zhou L Xu J Wang S Yang X Li C Zhou J. Papillary renal neoplasm with reverse polarity: A clinicopathologic study of 7 cases. Int J Surg Pathol (2020) 28(7):728–34. doi: 10.1177/1066896920918289 32403965

[5] Kim SS Cho YM Kim GH Kee KH Kim HS Kim KM. Recurrent KRAS mutations identified in papillary renal neoplasm with reverse polarity-a comparative study with papillary renal cell carcinoma. Mod Pathol (2020) 33(4):690–9. doi: 10.1038/s41379-019-0420-8 31953522

[6] Lee HJ Shin DH Park JY Kim SY Hwang CS Lee JH. Unilateral synchronous papillary renal neoplasm with reverse polarity and clear cell renal cell carcinoma: A case report with KRAS and PIK3CA mutations. Diagn Pathol (2020) 15(1):123. doi: 10.1186/s13000-020-01042-7 33023600 PMC7539524

[7] Song XX Xu ZY Ding XQ Chen ZK. Papillary renal tumor with polar inversion: Report of a case. Chin J Pathol (2020) 49(4):358–60. doi: 10.3760/cma.j.cn112151-20191016-00566 32268674

[8] Kiyozawa D Kohashi K Takamatsu D Yamamoto T Eto M Iwasaki T. Morphological, immunohistochemical, and genomic analyses of papillary renal neoplasm with reverse polarity. Hum Pathol (2021) 112:48–58. doi: 10.1016/j.humpath.2021.03.009 33811832

[9] Pivovarcikova K Grossmann P Hajkova V Alaghehbandan R Pitra T Perez Montiel D. Renal cell carcinomas with tubulopapillary architecture and oncocytic cells: Molecular analysis of 39 difficult tumors to classify. Ann Diagn Pathol (2021) 52:151734. doi: 10.1016/j.anndiagpath.2021.151734 33838490

[10] Wei S Kutikov A Patchefsky AS Flieder DB Talarchek JN Al-Saleem T. Papillary renal neoplasm with reverse polarity is often cystic: Report of 7 cases and review of 93 cases in the literature. Am J Surg Pathol (2022) 46(3):336–43. doi: 10.1097/PAS.0000000000001773 34352808

[11] Zhang GP Zhang YJ. Papillary renal neoplasm with reverse polarity: A case report. Asian J Surg (2021) 44(12):1606–7. doi: 10.1016/j.asjsur.2021.08.004 34635419

[12] Wang X Mao X Zhao Y Zhang Y. Papillary renal neoplasm with reverse polarity: A case report. Asian J Surg (2022) 45(11):2390–1. doi: 10.1016/j.asjsur.2022.05.166 35701276

[13] Delahunt B Cheville JC Martignoni G Humphrey PA Magi-Galluzzi C McKenney J. The international society of urological pathology (ISUP) grading system for renal cell carcinoma and other prognostic parameters. Am J Surg Pathol (2013) 37(10):1490–504. doi: 10.1097/PAS.0b013e318299f0fb 24025520

[14] Allory Y Ouazana D Boucher E Thiounn N Vieillefond A. Papillary renal cell carcinoma. Prognostic value of morphological subtypes in a clinicopathologic study of 43 cases. Virchows Arch (2003) 442(4):33642. doi: 10.1007/s00428-003-0787-1 12684768

[15] Lefèvre M Couturier J Sibony M Bazille C Boyer K Callard P. Adult papillary renal tumor with oncocytic cells: Clinicopathologic, immunohistochemical, and cytogenetic features of 10 cases. Am J Surg Pathol (2005) 29(12):157681. doi: 10.1097/01.pas.0000184821.09871.ec 16327429

[16] Saleeb RM Brimo F Farag M Rompré-Brodeur A Rotondo F Beharry V. Toward biological subtyping of papillary renal cell carcinoma with clinical implications through histologic, immunohistochemical, and molecular analysis. Am J Surg Pathol (2017) 41(12):161829. doi: 10.1097/PAS.0000000000000962 28984673

[17] Al-Obaidy KI Eble JN Nassiri M Cheng L Eldomery MK Williamson SR. Recurrent KRAS mutations in papillary renal neoplasm with reverse polarity. Mod Pathol (2020) 33(6):1157–64. doi: 10.1038/s41379-019-0362-1 31534204

[18] Gray RE Harris GT. Renal cell carcinoma: Diagnosis and management. Am Fam Physician (2019) 99(3):179–84. 30702258

[19] Khalifeh A Autorino R Eyraud R Samarasekera D Laydner H Panumatrassamee K. Three-year oncologic and renal functional outcomes after robot-assisted partial nephrectomy. Eur Urol (2013) 64(5):744–50. doi: 10.1016/j.eururo.2013.03.052 23639721

[20] Capitanio U Terrone C Antonelli A Minervini A Volpe A Furlan M. Nephron-sparing techniques independently decrease the risk of cardiovascular events relative to radical nephrectomy in patients with a T1a-T1b renal mass and normal preoperative renal function. Eur Urol (2015) 67(4):683–9. doi: 10.1016/j.eururo.2014.09.027 25282367

[21] Bhindi B Lohse CM Schulte PJ Mason RJ Cheville JC Boorjian SA. Predicting renal function outcomes after partial and radical nephrectomy. Eur Urol (2019) 75(5):766–72. doi: 10.1016/j.eururo.2018.11.021 30477983

[22] MacLennan S Imamura M Lapitan MC Omar MI Lam TB Hilvano-Cabungcal AM. Systematic review of perioperative and quality-of-life outcomes following surgical management of localised renal cancer. Eur Urol (2012) 62(6):1097–117. doi: 10.1016/j.eururo.2012.07.028 22841673

[23] Moch H Cubilla AL Humphrey PA Reuter VE Ulbright TM. The 2016 WHO classification of tumours of the urinary system and male genital organs-part a: Renal, penile, and testicular tumours. Eur Urol (2016) 70(1):93–105. doi: 10.1016/j.eururo.2016.02.029 26935559

[24] Moch H Amin MB Berney DM Compérat EM Gill AJ Hartmann A. The 2022 world health organization classification of tumours of the urinary system and male genital organs-part a: Renal, penile, and testicular tumours. Eur Urol (2022) 82(5):458–68. doi: 10.1016/j.eururo.2022.06.016 35853783

